# Intimal metastasis of undifferentiated pleomorphic sarcoma on the right chest wall mimicking an aortic embolus

**DOI:** 10.1093/ehjcr/ytae536

**Published:** 2024-09-26

**Authors:** Kosuke Sakai, Morihiro Higashi, Kazutsugu Uematsu

**Affiliations:** Department of Pulmonary Medicine, Saitama Medical Center, Saitama Medical University, 1981 Kamoda, Kawagoe-shi, Saitama 350-8550, Japan; Department of Pathology, Saitama Medical Center, Saitama Medical University, 1981 Kamoda, Kawagoe-shi, Saitama 350-8550, Japan; Department of Pulmonary Medicine, Saitama Medical Center, Saitama Medical University, 1981 Kamoda, Kawagoe-shi, Saitama 350-8550, Japan

**Figure:**
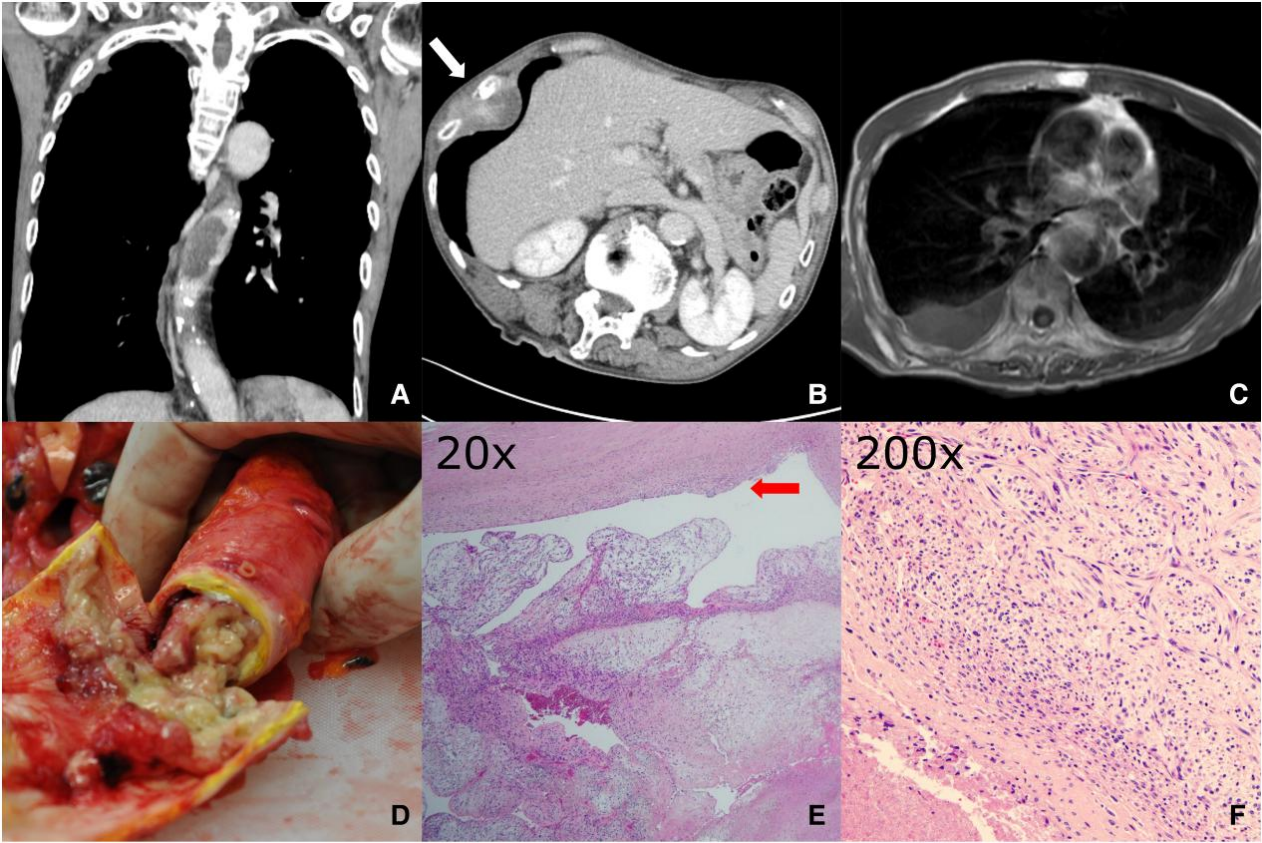


An 86-year-old man visited our hospital with right abdominal pain for 2 weeks. Contrast-enhanced computed tomography revealed a non-enhancing structure spanning > 55 mm in the thoracic descending aorta between the seventh and ninth thoracic vertebral levels (*Panel A*). Additionally, a heterogeneously enhanced mass (diameter, 65 mm) was observed on the right chest wall involving the eighth and ninth ribs (*Panel B*, white arrow). The intra-aortic structure was hyperintense on T1-weighted magnetic resonance images, indicating a thrombus in the descending aorta (*Panel C*). Needle biopsy of the chest wall lesion suggested spindle-cell carcinoma. The patient succumbed to disease progression 1 month after the initial visit, and an autopsy was performed. The right chest wall tumour was diagnosed as undifferentiated pleomorphic sarcoma (UPS), previously known as malignant fibrous histiocytoma. Atherosclerosis affected the aorta, extending from the thorax to the abdomen. In the descending thoracic aorta, a yellowish-white soft lesion occupied the lumen for >90 mm (*Panel D*), consisting of tumour emboli of proliferating UPS cells and necrosis (*Panels E*; red arrow, the aortic wall; *Panel F*). The tumour lesions extended from the vascular intima that was affected by the atheroma, and the elastic fibres in the tunica media were ruptured or missing. Although intimal metastases in the aorta have rarely been reported, possibly due to the rapid blood flow, factors affecting intimal conditions, such as arteriosclerosis, may promote intimal attachment of malignant cells derived from other organs.

(*A*) Coronal section of the chest contrast-enhanced computed tomography. (*B*) Transverse section of the chest contrast-enhanced computed tomography. The primary tumour is heterogeneously enhanced (white arrow). (*C*) Transverse section of the chest T1-weighted magnetic resonance image. (*D*) The gross examination of the lumen of the descending aorta. (*E*) The pathological finding of the lumen of the descending aorta (haematoxylin and eosin staining: 20×). The blood vessel is indicated by red arrow. (*F*) The pathological finding of the intravascular metastasis of the undifferentiated pleomorphic sarcoma with necrosis (haematoxylin and eosin: 200×).

## Data Availability

The data underlying this article are available from the corresponding author upon reasonable request.

## Notes

**Consent:** All Authors have approved the final version of the manuscript for publication and agreed to be accountable for all aspects of this work. The kin of the deceased patient provided written consent for the use of all data for academic and research purposes, including the publication of case studies and images.

**Funding:** This study did not receive any financial support or funding.

